# The Effect of Heating Rate on the Microstructure Evolution and Hardness of Heterogeneous Manganese Steel

**DOI:** 10.3390/ma17215321

**Published:** 2024-10-31

**Authors:** Wubin Ren, Peiyu Zhao, Menghu Wang, Shuai Tong, Xiaokai Liang, Xinjun Sun, Huibin Wu

**Affiliations:** 1 Engineering Steel Institute, Central Iron and Steel Research Institute, Beijing 100081, China; d202410672@xs.ustb.edu.cn (W.R.); wang.mh98@foxmail.com (M.W.); tongshuai@cisri.com.cn (S.T.); liangxk@sina.com (X.L.); 2 Collaborative Innovation Center of Steel Technology, University of Science and Technology Beijing, Beijing 100083, China; whbustb@163.com; 3 Construction Project Management Branch, China Oil and Gas Piping Networking, Langfang 065000, China; zhaopy01@pipechina.com.cn

**Keywords:** microstructure, hardness, heating rate, retained austenite, ghost pearlite

## Abstract

The use of a rapid heating method to achieve heterogeneity of Mn in medium-manganese steel and improve its comprehensive performance has been widely studied and these techniques have been widely applied. However, the heating rate (from α to γ) has not received sufficient attention with respect to its microstructure-evolution mechanism. In this study, the effect of heating rate on the microstructure evolution and hardness of heterogeneous medium-manganese steel was investigated by using X-ray diffraction (XRD), scanning electron microscopy (SEM), electron backscatter diffraction (EBSD), transmission electron microscopy (TEM) and DICTRA simulation. The results showed that the Mn distribution was heterogeneous in the initial microstructure of pearlite due to strong partitioning of Mn between ferrite and cementite. At low heating rates (<10 °C/s), the heterogeneity of Mn distribution was diminished to some extent due to the long-distance diffusion of Mn in high-temperature austenite. Contrastingly, at high heating rates, the initial heterogeneity of the Mn element could be largely preserved due to insufficient diffusion of Mn, which resulted in more ghost pearlite (GP: pearlite-like microstructure with film martensite/RA). Moreover, the high heating rate not only refines the prior austenite grain but also increases the total RA content, which is mainly composed of additional film RA. As the heating rate increases, the hardness gradually increases from 628.1 HV to 663.3 HV, due to grain refinement and increased dislocation density. Dynamic simulations have also demonstrated a strong correlation between this interesting microstructure and the non-equilibrium diffusion of Mn.

## 1. Introduction

Medium-manganese steel, possessing high strength, high plasticity and excellent work-hardening ability, is widely used in various fields such as automobile manufacturing, wear-resistant component manufacturing and offshore platform construction [1,2,3,4]. It has been found that its excellent comprehensive mechanical properties are mainly due to the transformation-induced plasticity (TRIP) effect of retained austenite (RA) in the deformation process, which greatly improves the plasticity of the material [5,6]. The proportion and morphology of the RA have an important impact on the overall mechanical properties, as indicated in References [7,8]. Moreover, Zhang et al. [9,10] have demonstrated that an increased fraction of RA contributes to enhanced total elongation of the material. Also, the formation of film RA ensures a larger uniform elongation, thereby obtaining good overall and local plasticity. Based on these findings, Li et al. [11] employed the austenite reverted transformation (ART) process to treat 0.2C-1.6Al-6.1Mn hot-rolled medium-manganese steel. Levels of more than 25% RA were obtained, successfully achieving a good balance between strength and plasticity. Therefore, regulating the morphology and proportion of the RA plays a significant role in improving the comprehensive mechanical properties of medium-manganese steel.

Rapid heating technology has received widespread attention due to its various advantages, including simplifying production processes, improving efficiency, and being environmentally friendly and low-carbon [12,13,14,15]. The application of rapid heating to heterogeneous microstructures often leads to advancements in properties. For instance, a microstructure containing 18.5% RA was obtained by the rapid heating and short-term insulation treatment of 0.51C-4.35Mn steel [16]. Ding et al. [17] demonstrated the generation of Mn-rich RA and Mn-poor ferrite through rapid heating of ART-treated 0.18C-8.0Mn steel. This process produced a heterogeneous distribution of Mn within high-temperature austenite, ultimately resulting in a strength exceeding 2 GPa and an elongation exceeding 20%. Kim et al. [18] obtained heterogeneous microstructures with different Mn distributions in 0.18C-3.5Mn-0.1Si steel by annealing at 660 °C for 24 h. Through subsequent rapid heating and quenching, a synchronous improvement of strength and plasticity was achieved. Therefore, it is expected that the strength and plasticity of steel can be improved simultaneously by designing a chemically heterogeneous morphology combined with a rapid heating process.

In recent studies, it has been found that reductions in the manganese content in medium-manganese steel, combined with chemical inhomogeneity and flash heating technology, improve the yield strength of the material, while also reducing the Lüders band phenomenon [19]. Moreover, it was found that the microstructure of hot-rolled medium-manganese steel changed after ultrafast heating treatment, including austenite recrystallization, carbon atom diffusion and dislocation rearrangement [20]. However, it was also found that reducing the heating rate can improve the mechanical properties and ductility of medium-manganese steel by optimizing its manganese distribution [21]. A large number of studies have found that the mechanical properties of medium-manganese steel can be improved by rapid heating, but the heating rate (from α to γ) has not received sufficient attention, specifically as to its microstructure evolution mechanism [22,23,24].

Due to the short duration of high temperature, the rapid heating process significantly affects the diffusion of elements and the evolution of microstructure. Studying the effect of heating rate on its microstructure and mechanical property can help in the selection of the appropriate heating processes for practical production. Therefore, a 0.4C-5Mn medium-manganese steel was designed in this work. The heterogeneous microstructure of laminate pearlite was obtained by pretreatment. On this basis, the microstructure evolution and element distribution under different heating rates (0.3–200 °C/s) were studied, an inquiry which may provide more guidance for the development of medium-manganese steel.

## 2. Materials and Experimental Procedures

**Figure 1:**
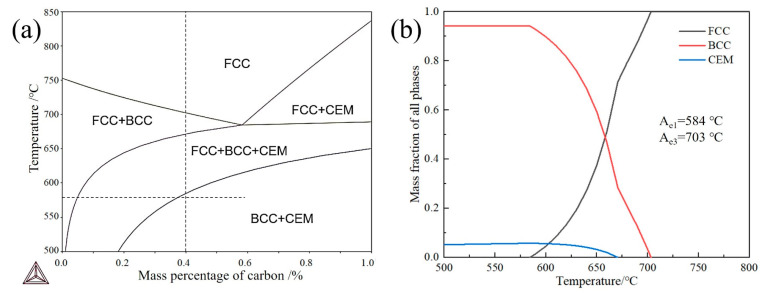
Calculation results using the TCFE10 database by Thermo-Calc software: (**a**) phase diagram, in which vertical dotted lines represent the chemical composition of the designed steel; (**b**) step diagram.

**Figure 2:**
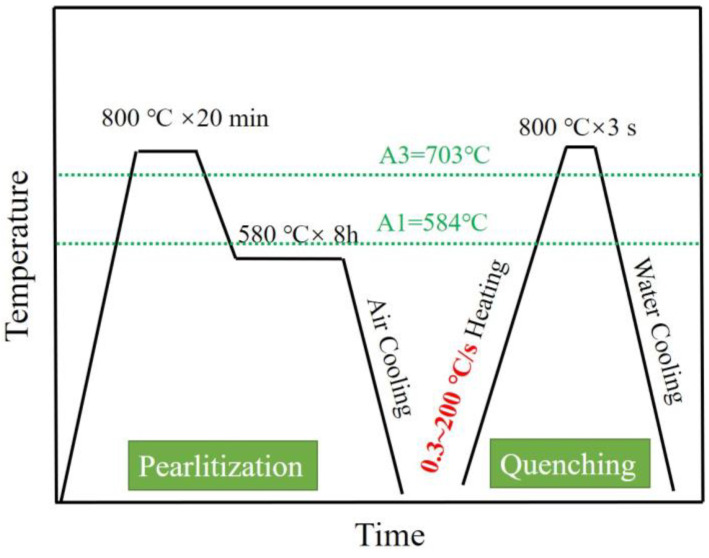
Pearlite pretreatment and quenching process after heating of the tested steels at different rates.

The 0.4C-5Mn medium-manganese steel was melted by a 200 kg vacuum induction furnace under an argon atmosphere. It was initially forged into a billet measuring 250 mm × 150 mm × 60 mm. The billet was homogenized at 1050 °C for 2 h, and then hot-rolled into a thickness of 10 mm with seven passes. The finish rolling temperature was maintained at 780 °C, and this was followed by air cooling. The chemical composition of the tested steel is listed in Table 1. The phase diagram and step diagram of the steel were calculated using Thermo-calc software (2023a) with the TCFE10 database, as shown in Figure 1. The starting and ending transformation temperatures of austenite are 584 °C and 703 °C, respectively. In accord with the requirement of isothermal transformation in the two-phase region of ferrite and cementite, a pre-treatment temperature of 580 °C for the pearlite transformation was selected. Firstly, the hot-rolled steel plate was heated to 800 °C for 20 min. It was then heated it to 580 °C for 8 h to achieve pearlitization; this was followed by air cooling. Subsequently, samples of 8 mm × 12 mm were cut for Gleeble thermal simulation testing. The model of the thermal simulation testing machine used is the Gleeble 3800, with a thermocouple wire connected in the middle of the sample. After completing the set process, samples were immediately thrown into water and quenched. The samples were heated at rates of 0.3, 1, 3, 10, 30, 50, 100, and 200 °C/s to 800 °C; holding for 3 s followed, and then the samples were immediately quenched to room temperature. The complete heat treatment process is shown in Figure 2; the process is called pearlite partitioning quenching (PPQ).

Before the X-ray diffraction (XRD) test, the sample is polished, and the surface stress is removed by electrolysis. The Co target was installed using a German Brooke D8 ADVANCE X-ray diffractometer for XRD testing, with a scanning speed of 2°/min and a scanning angle of 2θ, ranging from 45° to 115°. Jade6.5 software was used to process the raw XRD data. The volume fraction of phase was estimated using the following expression [25]:

(1) $$V_{i}=\frac{\frac{1}{n}\sum_{i=1}^{n}\frac{I_{i}^{j}}{R_{i}^{j}}}{\frac{1}{n}\sum_{j=1}^{n}\frac{I_{\alpha }^{j}}{R_{\alpha }^{j}}+\frac{1}{n}\sum_{j=1}^{n}\frac{I_{i}^{j}}{R_{i}^{j}}}$$

where *i* is phase types; *n* and $I_{i}^{j}$ are the number and integrated intensity of diffraction peaks; and $R_{i}^{j}$ is the material scattering coefficient. For microstructure analysis, the sample was polished and etched using a 4% nitric acid alcohol solution, and the microstructure was observed using an FEI Quanta 650 scanning electron microscope (SEM). For the observation of the electron backscatter diffraction (EBSD) microstructure, a JSM-7900F field emission scanning electron microscope equipped with an Oxford F-plus backscatter diffractometer was used. The scanning area was 45.0 μm × 27.5 μm, with a step of 0.035 μm. The data were processed using HKL Channel 5 software. After mechanical polishing, the EBSD samples were subjected to vibration polishing using a Buehler Vibromet 2 vibration polishing instrument with a silica suspension as the polishing solution. To prepare the samples for transmission electron microscopy (TEM), slices of 300 μm thickness were cut from the sample, then thinned down to 50 μm thickness, and finally punched into round disks with 3 mm diameters. Subsequently, the disks were further thinned using a twin-jet electrolytic polisher. Observation and elemental testing of the disks were performed using Talos F200X G2 transmission electron microscopy equipped with EDS spectroscopy. The Mn content (*U*_Mn_) was calculated by Mn/(Mn + Fe + Si + Mo), where Mn, Fe, Si, and Mo represented their atomic fractions.

The model of the digital Vickers hardness tester used is the VH-5, with a load of 10 kg and a time of 15 s. The data were measured by EA-2A measurement software. At least 5 points were tested on the samples under each process, and the average value was taken as the final hardness result. An EM500-2A semi-automatic micro Vickers hardness tester was used to test the microhardness of different phases, with a load of 0.05 kg and a time of 10 s. Each sample was tested at no less than 5 points, and the average value was taken as the final result. The distance between any two test center points must be no less than 2.5 times the diameter of the indentation.

## 3. Results

### 3.1. Microstructure Characterization

#### 3.1.1. Initial Microstructure Characterization

**Figure 3:**
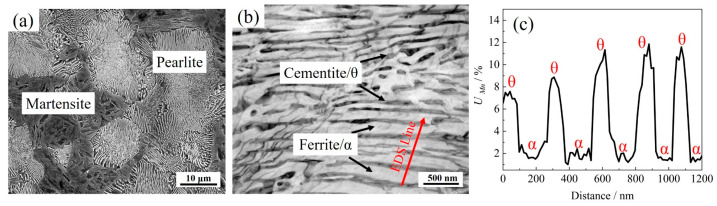
(**a**) SEM characterization of initial microstructure and (**b**) TEM characterization of pearlite. (**c**) Mn profiles along the scanning line in (**b**).

Figure 3a shows the microstructure of the tested steel after insulation treatment at 580 °C for 8 h, mainly composed of pearlite (74.1 ± 1.2%) and martensite. Figure 3b shows the fine structure of pearlite, consisting of black cementite and white ferrite. The energy spectrum scanning results show that the Mn content in the cementite is about 8.87%, significantly higher than that in the ferrite (about 1.93%), as shown in Figure 3c. This is due to strong Mn partitioning between the two phases [26]. The interlayer spacing of pearlite is about 182.5 nm, and the width of ferrite is significantly larger than that of cementite.

#### 3.1.2. Prior Austenite Grains

**Figure 4:**
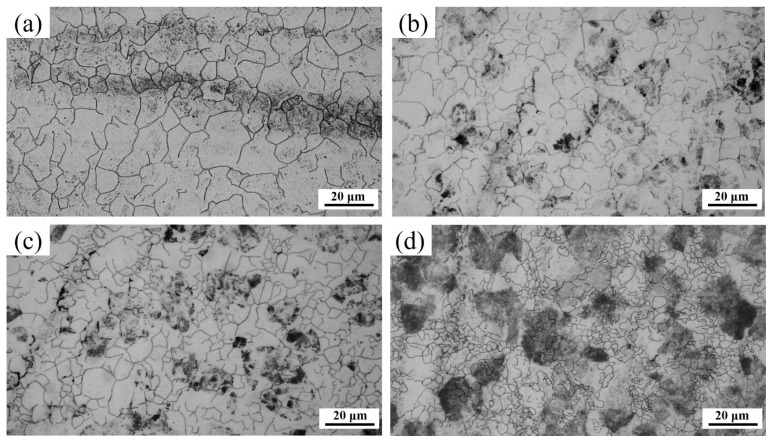
Prior austenite grains at different heating rates: (**a**) 0.3 °C/s, (**b**) 10 °C/s, (**c**) 50 °C/s, and (**d**) 200 °C/s.

**Figure 5:**
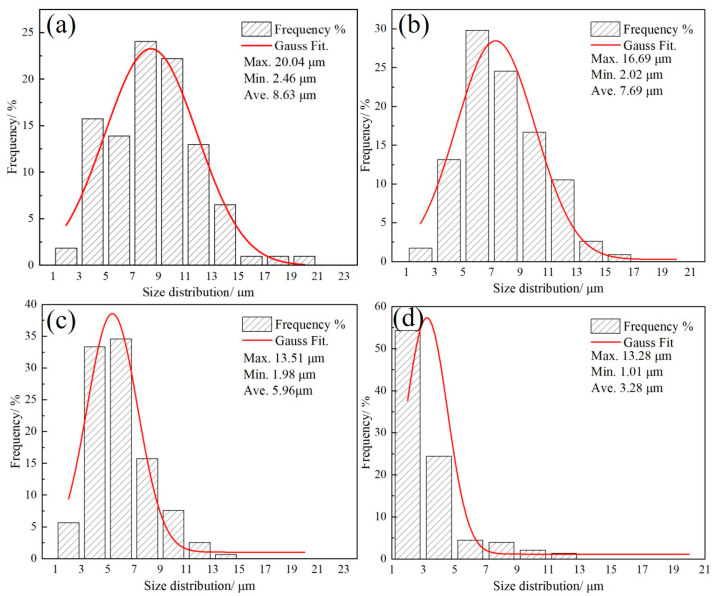
Distribution statistics of PAGs at different heating rates: (**a**) 0.3 °C/s, (**b**) 10 °C/s, (**c**) 50 °C/s, and (**d**) 200 °C/s.

Figure 4 shows the prior austenite grains (PAGs) at different heating rates, in which the black phase is caused by severe element inhomogeneity. When the heating rate is 0.3 °C/s, the average austenite grain size is 8.63 μm. Furthermore, it is apparent that the sizes of the PAGs decrease with an increase in heating rate, relative to which the sizes of the PAGs decrease approximately 38% between the heating rates of 0.3 °C/s to 200 °C/s. It was found that when the heating rate exceeds 10 °C/s, the black microstructure area becomes obvious, which may be caused by uneven corrosion caused by local element aggregation. Figure 5 shows the detailed distribution of the PAGs at different heating rates. It was determined that the sizes of both the maximum and minimum grains decrease with the increase in heating rate. More importantly, the grain size of the PAGs becomes more concentrated. The refined and concentrated PAGs are beneficial for the uniformity of microstructure. This is because the small initial PAGs are obtained at a high heating rate, subject to which only a few grains tend to grow, as the austenitization time shortens before the PAGs flatten [15].

Previous studies have suggested that medium-manganese steel has strong structural heritability, meaning that high heating rates cannot refine PAGs [27]. Because RA in the original microstructure becomes the nucleation source of austenite, austenite will inherit the orientation growth of the RA. Many nucleation sources belonging to the same PAG will grow in the same direction. Eventually, as the grain boundaries continue to grow, they will merge and disappear, forming a new PAG of the same size as the old PAG. In this study, RA was eliminated through the pearlite pretreatment. Rapid heating promotes the nucleation density of austenite. Additional austenite sources will grow in different directions, thereby refining the PAGs.

#### 3.1.3. Microstructure Analysis of RA

**Figure 6:**
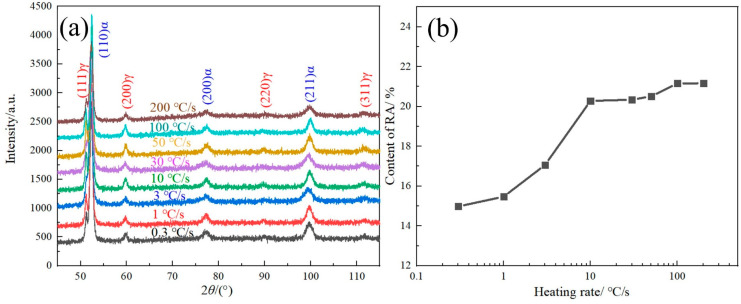
XRD pattern (**a**) and RA content variation curve with heating rate (**b**).

The XRD patterns of the tested steels with different heating rates are shown in Figure 6a, in which obvious austenite diffraction peaks are observed. The content of RA is shown in Figure 6b, as calculated using Equation (1). As the heating rate increases from 0.3 °C/s to 10 °C/s, the content of RA gradually increases from 14.98% to 20.28%. However, as the heating rate further increases (>10 °C/s), the content of RA remains basically unchanged, rising from 20.28% to 21.17%. This is because the heating rate affects the distribution of the Mn element, thereby affecting the content of the RA. The Mn element can expand the austenite phase and improve the stability of austenite, so an aggregation of Mn would be beneficial for increasing the content of RA.

**Figure 7:**
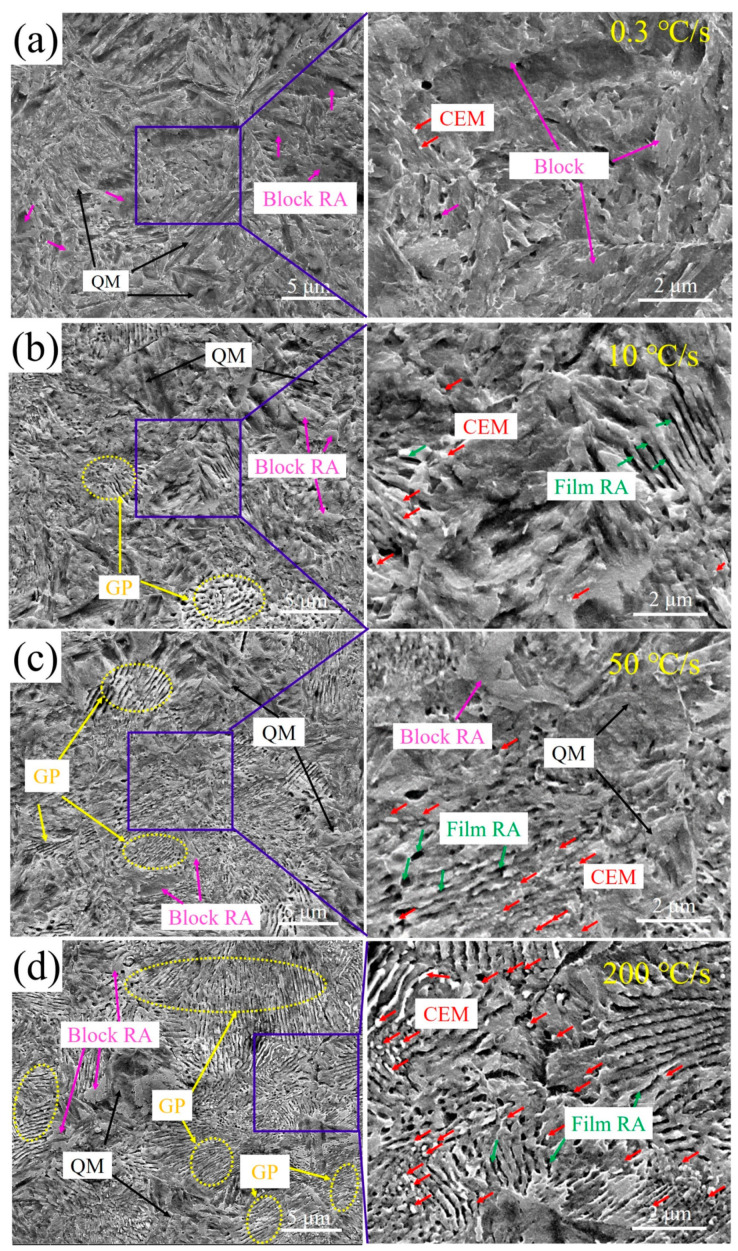
SEM microstructure at different heating rates: (**a**) 0.3 °C/s, (**b**) 10 °C/s, (**c**) 50 °C/s, and (**d**) 200 °C/s.

Due to the initial microstructure, which includes martensite and pearlite, the microstructure becomes more complex after reverse phase transformation. The SEM microstructure of the tested steels with different heating rates includes quenched martensite (QM), ghost pearlite (GP), RA and cementite (CEM), all of which are marked in Figure 7. Furthermore, the morphology of RA can be classified into block shape and film forms. At a heating rate of 0.3 °C/s, the microstructure is mainly composed of QM and a certain amount of block RA, with a very low content of undissolved CEM (Figure 7a). The slow heating rate allows for the gradual homogenization of Mn in the initial pearlite, reducing the stability of the high-temperature austenite and making it more susceptible to transforming into martensite during quenching. Consequently, the martensite content is the highest at this rate. At a heating rate of 10 °C/s, the content of GP (pearlite-like microstructure with film martensite/RA) significantly increases. The black phase in GP is film RA (Figure 7b), which also increases with the increase of GP. However, an increased amount of CEM, respectively indicated by red arrows, is undissolved. This is due to the low austenitization temperature (800 °C) and short holding time (3 s). When the heating rate is 50 °C/s, GP does not significantly increase, which leads to a slight increasement of the content RA. However, the microstructure becomes finer. Additionally, when the heating rate is 200 °C/s, the GP morphology is most obvious (Figure 7d). Meanwhile, the microstructure is further refined.

**Figure 8:**
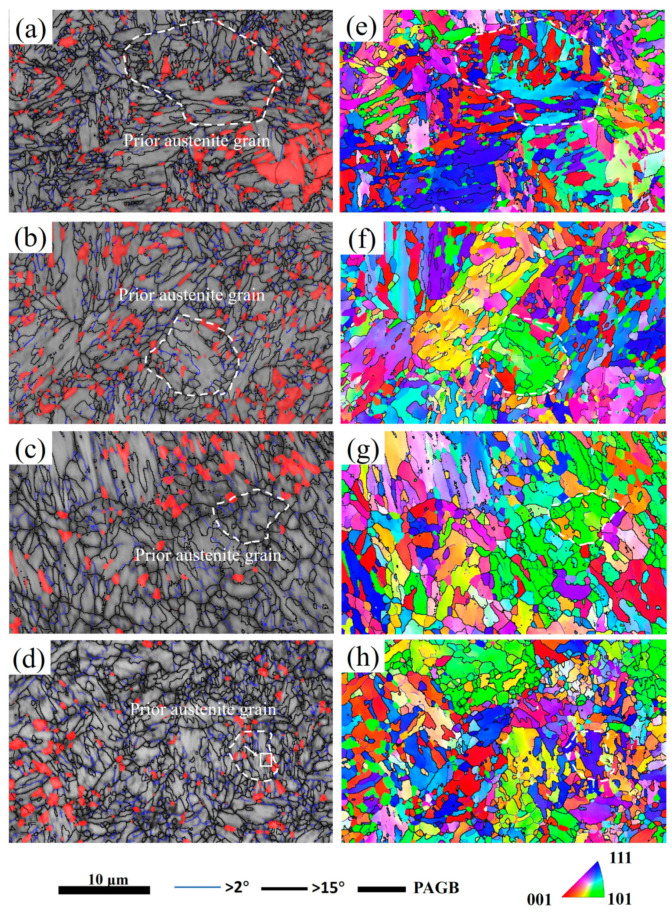
EBSD phase distribution of the tested steels (**a**–**d**) after heating at different rates; the red phase is RA, and the gray phase is martensite: IPF diagram (**e**–**h**); (**a**,**e**) 0.3 °C/s; (**b**,**f**) 10 °C/s; (**c**,**g**) 50 °C/s; and (**d**,**h**) 200 °C/s.

The EBSD microstructure of the tested steels is shown in Figure 8. The yellow blocks in Figure 8 are prior austenite grains. As the heating rate increases, PAGs gradually refine, which is consistent with the results reflected in Figure 4. As the heating rate increases, the content of block RA first slightly increases and then decreases. When the heating rate was 3 °C/s, a distinctive characteristic of lath martensite was observed (Figure 8a). However, with the increase in the heating rate, the characteristics of lath martensite gradually disappeared (Figure 8c,d). This indicates that high heating rates will promote non-equilibrium reverse phase transformation, forming non-uniform microstructures at high temperatures. The IPF plots of Figure 8e–h showed that the RA within the same PAG has a similar orientation and almost no internal substructure. The RA content in Figure 8a–d is significantly lower than determined in the results of XRD testing, which is limited by the resolution of EBSD technology, resulting in the film RA not being recognized.

**Figure 9:**
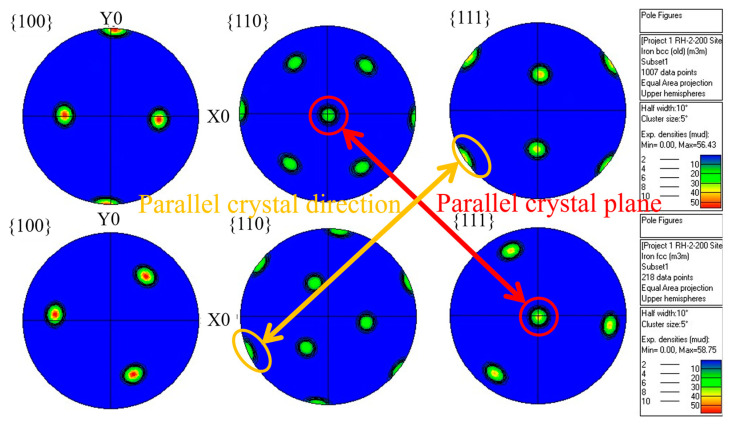
Polar diagram of the martensite and austenite within the selected white box in Figure 8d.

The local area in Figure 8d was extracted, and a polar diagram for block RA and QM phases was created, in which the crystal coordinate system needed to be rotated to bring it closer to the standard polar diagram (as shown in Figure 9). According to the principles of equatorial plane projection, a crystal plane parallel to the equatorial plane projects at the center of the polar graph. Conversely, a crystal plane perpendicular to the equatorial plane projects at the edge of the polar graph. Therefore, the block RA formed by rapid heating of the tested steels satisfies the following crystallographic orientation relationship with the matrix martensite:

(2) $${\left\{110\right\}}_{M}∥{\left\{111\right\}}_{\gamma };<111{>}_{M}∥<110{>}_{\gamma }$$

**Figure 10:**
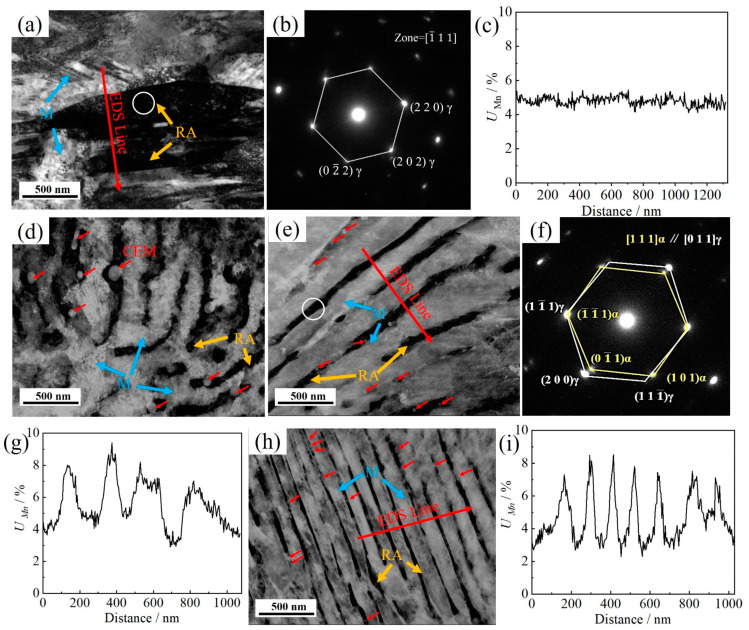
TEM microstructure characterization of the tested steels heated at a rate of 0.3~200 °C/s: bright field image after heating at 0.3 °C/s (**a**), 10 °C/s (**d**), 50 °C/s (**e**), and 200 °C/s (**h**). Images (**b**,**f**) are the SAED patterns from the white circles in (**a**,**e**), respectively; images (**c**,**g**,**i**) are the energy spectra of the Mn content in the red-lined area in (**a**,**e**,**h**), respectively.

Figure 10 shows the bright field image, selected area electron diffraction (SAED) patterns, and line distribution of Mn for the multiphase microstructure. When the heating rate is 0.3 °C/s, a block RA of several hundred nanometers is observed, as shown in Figure 10a. As shown in Figure 10c, a uniform distribution of Mn was observed across the blocky RA and the martensite. When heated at 10 °C/s, an alternating structure of RA and lath martensite is observed. More spherical or rod-shaped undissolved CEM are distributed on the film RA. The SAED pattern indicates that both RA and lath martensite adhere to the K-S orientation relationship (Figure 10d). As the heating rate is further increased to 50 °C/s, the phase boundary between RA and lath martensite becomes more linear. These two phases still maintain the K-S orientation relationship (Figure 10f). The EDS analysis reveals that RA is enriched in Mn (7.14 ± 0.31%), whereas lath martensite is poor in Mn (3.81 ± 0.14%), as shown in Figure 10g. During quenching stage, the Mn-poor austenite transforms into martensite along the habitual plane of {111}_γ_. Conversely, the adjacent Mn-enriched austenite, with higher thermal stability, does not transform into martensite, ultimately being retained as RA. Thus, the phase boundary is consistent with the chemical boundary of the Mn element. When heated at 200 °C/s, the enrichment of Mn in RA further increases (7.21 ± 0.24%), and the deficiency of Mn in martensite becomes more pronounced (3.40 ± 0.18%). Consequently, the high heating rate establishes a distinct chemical boundary dominated by Mn, forcing the transformation of martensite into a smaller area and ultimately leading to the formation of an extremely fine microstructure. At this rate, the average width of the film martensite is 159.1 nm, corresponding to an average width of 19.7 nm for RA. The interlayer spacing of GP is substantially equivalent to the spacing of the initial pearlite.

#### 3.1.4. Dislocation Density

**Figure 11:**
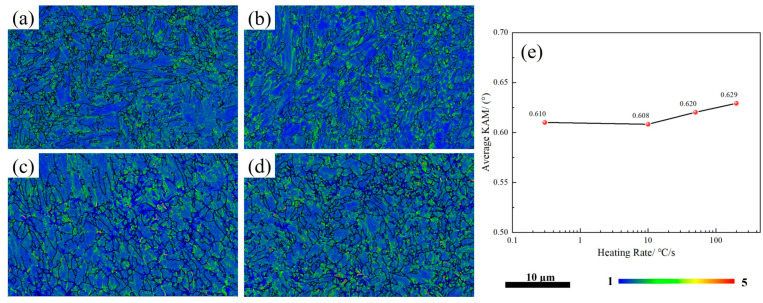
KAM diagrams and average KAM values of the tested steels heated at different rates: (**a**) 0.3 °C/s, (**b**) 10 °C/s, (**c**) 50 °C/s, (**d**) 200 °C/s, and (**e**) average KAM values.

The kernel average misorientation (KAM) diagram is capable of measuring the local orientation gradient resulting from deformation within the grain. Moreover, the local residual strain in the lattice is associated with the local orientation difference in the microstructure. Figure 11 shows the KAM diagrams and corresponding average KAM values after heating at different rates. From Figure 8a–d and Figure 11a–d, it can be observed that higher KAM is mainly distributed within the martensite, while lower KAM is found within the blocky RA. Moreover, regions with smaller grains were found to have higher KAM values. As the heating rate increases, the KAM value first decreases and then gradually increases, but the change is not significant. The highest average KAM value of the tested steel heated at 200 °C may be related to its lower block RA content and smaller grain size. The value of dislocation density can be estimated using Equation (3) [28]:

(3) ρ≅2θ/μb

where *θ* is the low angle orientation difference (KAM value), u is the step size of the EBSD scan (350 nm), and b is the Burgers vector size (taken as 0.248 nm). After calculation, the lowest dislocation density, for the tested steel heated at 10 °C/s, is 1.4009 × 10^12^ cm^−2^, and the highest dislocation density, for the tested steel heated at 200 °C/s, is 1.4493 × 10^12^ cm^−2^.

Considering the step accuracy and regional limitations of EBSD technology, XRD results were used to further calculate dislocation density. The relationship between the X-ray peak width and dislocation density is estimated by Equation (4) [29]:

(4) $$\Delta K=\frac{\alpha_{s}}{D}+\mathrm{Nb}\sqrt{\rho }K$$

where ΔK is the peak width, N is a constant (0.263), α_s_ is the deformation factor (0.9), D is the grain size, K is the diffraction vector size, b is the Burgers vector, and ρ is the dislocation density. K is directly proportional to the diffraction angle θ and wavelength λ, as expressed by Equation (5):

(5) K=2sinθ/λ

By combining Equations (4) and (5), this can be further expressed as Equation (6):

(6) $$\Delta K=\frac{\alpha_{s}}{D}+\frac{2\sin\theta \mathrm{Nb}\sqrt{\rho })}{\lambda }$$

After calculation, the dislocation density of the tested steel heated at 1 °C/s is lowest, at 1.2855 × 10^12^ cm^−2^, and the dislocation density of the tested steel heated at 200 °C/s is highest, at 1.5332 × 10^12^ cm^−2^. Table 2 shows the dislocation density values measured by XRD under different heating rates. It can be observed that a high heating rate can increase dislocation density to some extent.

### 3.2. Hardness

**Figure 12:**
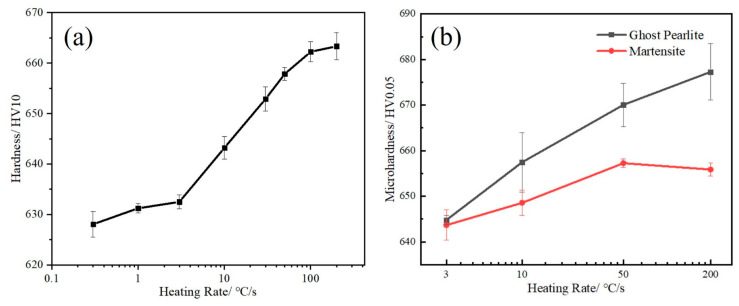
Vickers hardness and microhardness of test steels after heating at different rates. (**a**) Macro hardness variation curve with heating rate (**b**) The variation curve of microhardness of ghost pearlite and martensite with heating rate.

**Figure 13:**
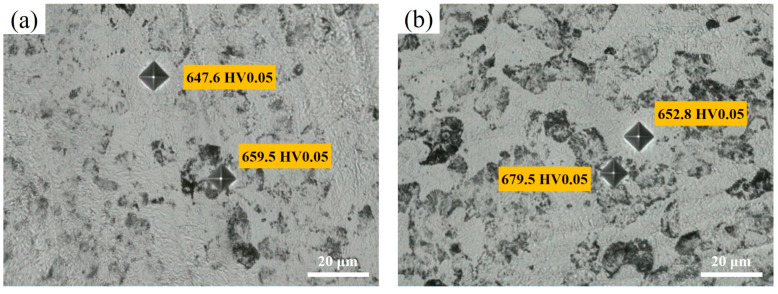
Microhardness indentation images of the tested steels heated at 10 °C/s (**a**) and 200 °C/s (**b**).

Figure 12a shows the hardness curves of the tested steels after heating at different rates. As the heating rate increases, the hardness increases from 628.1 HV to 663.3HV. It is interesting that the hardness increases slowly when the heating rate is below 3 °C/s. However, when the heating rate exceeds 10 °C/s, the hardness begins to increase rapidly. The hardness of the tested steel exceeds 660 HV10 when the heating rate exceeds 100 °C/s. The microhardness of the two microstructures in the experimental steels is shown in Figure 13b. The microhardness of ghost pearlite gradually increases with the increase in the heating rate, while the microhardness of martensite first increases and then decreases. Moreover, the microhardness of ghost pearlite is higher than that of martensite. Figure 13 shows the microhardness indentation images of two different microstructures. Under heating at 10 °C/s and 200 °C/s, the indentation size on the pearlite is significantly smaller than that on the martensite. The purpose of introducing chemical heterogeneity is to create micro-regions with different strength levels, which is a key factor in improving the strength of high-strength martensitic steels.

## 4. Discussion

### 4.1. The Influence of Heating Rate on RA Morphology

**Figure 14:**
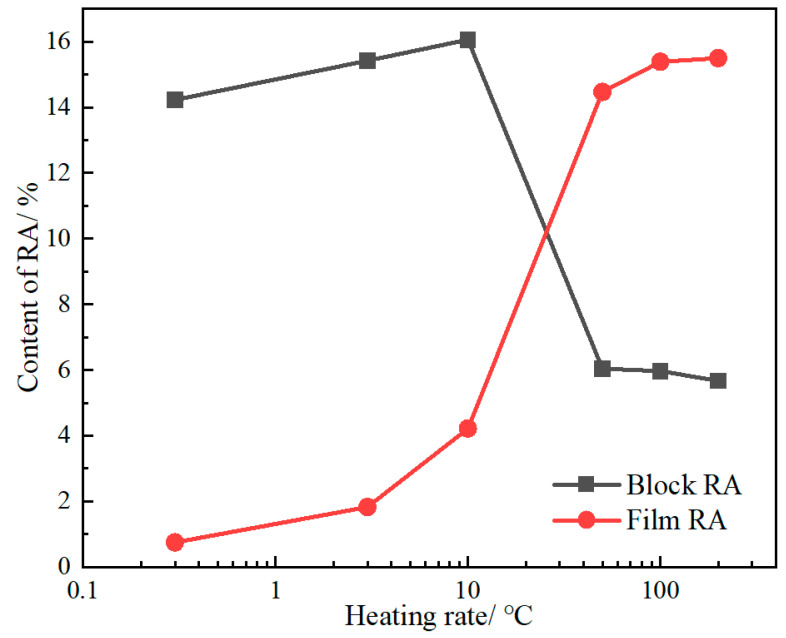
The volume fraction of film and blocky RA after heating at different rates.

The tested steels heated at different rates mainly consist of film RA and blocky RA, as shown in Figure 7. The content of block RA was manually estimated by SEM images using Photoshop software (CC 2017), and the content of total RA was measured using XRD results. Following this, the film RA fraction is deduced by taking total RA fraction and subtracting the block RA fraction. With an increase in the heating rate from 0.3 °C/s to 10 °C/s, the block and film RA both increase (Figure 14). This is because the increase in heating rate pushed the nucleation and growth process of austenite towards the high-temperature range. In a higher temperature range, the NPLE diffusion mechanism accelerates the growth of austenite, which will increase the fraction of block RA [30]. Furthermore, the content of block RA is much higher than that of film RA. Due to the presence of pearlite and martensite in the initial microstructure, the low heating rate leads to a certain degree of homogenization of elements in the high-temperature austenite, resulting in the production of a large amount of block RA in the GP region (derived from pearlite), as shown in Figure 7a,b.

With the heating rate increased from 10 °C/s to 50 °C/s, a rapid decline in blocky RA content was observed, accompanied by an accelerated surge in film RA content, as shown in Figure 7c,d. Under high heating rates, the chemical fluctuations of Mn are retained to a certain extent, which will promote the alternating distribution of microstructure (Figure 10e,h). This chemical pattern is inherited from partitioned pearlite consisting of Mn-enriched cementite and Mn-depleted ferrite (Figure 3b), which lead to the formation of alternative film RA and lath martensite after quenching to room temperature, respectively. When the heating rate exceeded 50 °C/s, the content of RA in both the film and the block remained basically unchanged. This indicated that the chemical heterogeneity retained sufficient potency to persist into room temperature range at this high heating rate. Further increases in the heating rate failed to proportionally increase the film RA content. However, a more concentrated distribution of Mn will refine the film RA and martensite (Figure 10h).

There are many factors that determine the stability of RA, with Ms temperature as the measurement index. It is calculated using the modified Mahieu’s Formula (7), which considers the influence of chemical compositions and grain size [31,32].

(7) $$M_{s}=539-423\chi_{C}-30.4\chi_{Mn}-7.5\chi_{Si}-56.5d^{-1/2}$$

In the formula, χ_C,_ χ_Mn_, and χ_Si_ represent the content of local austenite region C, Mn, and Si, respectively, and *d* represents the size of the RA region. Therefore, higher alloy element content (mainly C, Mn) and smaller grain size will improve the stability of RA. As shown in Figure 10g,i, with the increase in heating rate, the Mn content of the film RA increases and the size decreases, thus exhibiting higher stability. On the contrary, the Mn content of the block RA formed at low heating rates is basically the same as that of the matrix Mn content (Figure 10a), and the size is relatively large (Figure 7a and Figure 8a); it thus exhibits lower stability. It can be foreseen that this RA with different stability will enable the tested steels to balance higher total elongation and uniform elongation.

### 4.2. The Influence of Heating Rate on Hardness

By rapidly heating the medium-manganese steel with a martensite + pearlite duplex microstructure, an excellent combination of hardness exceeding 660 HV10 and RA fraction exceeding 20% was achieved. It is generally believed that RA as a soft phase will reduce the hardness of the material, but this study not only obtained more RA but also significantly improved the hardness, which is related to the heterogeneous microstructure. As shown in Figure 12, the higher-hardness ghost pearlite contributes more to improving the overall hardness. The chemical inhomogeneity of Mn still remains in the ghost pearlite, resulting in the formation of extremely fine film martensite lath and RA with alternating distribution (Figure 10e,h). The yield strength (*Y*_s_) and hardness (HV) can be converted using Equation (8) [33]:

(8) $$\sigma_{y}=-90.7+2.876HV$$

The yield strength of the tested steels can be formulated by using a modified form of the Hall–Petch Equation (9):

(9) $$\sigma_{y}=\sigma_{0}+k_{y}d^{-1/2}+\sigma_{ss}+\sqrt{{\sigma_{p}}^{2}+{\sigma_{d}}^{2}}$$

where *σ*_0_ is the intrinsic lattice friction stress for pure Fe, k_y_ is a constant, d is average grain size, *σ*_ss_ is the strength increment of solid solution hardening, *σ*_p_ represents the strength increment due to precipitation strengthening, and *σ*_d_ is the dislocation strengthening term.

In this work, the values of *σ*_0_ and k_y_ in Equation (9) are taken as 54 MPa and 365.5 MPa·μm^1/2^, respectively. As the heating rate increases, the average PAGs gradually refine, from a maximum of 8.63 μm to 3.28 μm. Therefore, the increment of fine-grain strengthening increased from 124.4 MPa to 201.8 MPa. Solid solution strengthening is related to the content of solid solution atoms, so it is necessary to remove the influence of solid solution atoms consumed by second-phase particles. Due to the high heating rate, the amount of undissolved second-phase particles increases, thereby reducing the solid solution content of the alloy.

The increment of solid solution strengthening can be calculated using Equation (10):

(10) $$\sigma_{ss}=4570[C]+470[P]+83[Si]+37[\mathrm{Mn}]+11[\mathrm{Mo}]$$

where [i] represents the weight fraction of solid solution element. For quenched martensitic steel, the increment of solid solution strengthening is mainly determined by the interstitial atoms C. With the increase in heating rate, the content of undissolved second phases (mainly alloy cementite) increases, which leads to a decrease in the solid solution carbon content. Furthermore, the dissolved carbon will fully diffuse into the high-temperature austenite during the 3 s insulation stage. Therefore, a high heating rate will reduce the increment of solid solution strengthening to some extent.

To estimate σ_p_, the Ashby Orowan equation was used, as described in the following [34]:

(11) $$\sigma_{p}=(\frac{0.538\mathrm{Gb}\sqrt{V_{f}}}{x})\ln(\frac{x}{2b})$$

Among these terms, G is the shear modulus, B is the Burgers vector, *V*_f_ is the volume fraction of the precipitate, and *x* is the average diameter of the precipitate. It is generally believed that the increase in yield strength of second-phase particles with a size greater than 60 nm is so small that it can be ignored. Through analysis of TEM observations, it can be determined that the second-phase particles are mainly alloy cementite (M3C) with a size exceeding 100 nm. Therefore, the strength improvement brought by the precipitation strengthening of the experimental steels is very limited and can be ignored here.

Dislocation hardening stress *σ_d_* can be estimated by the following equation:

(12) $$\sigma_{d}=M\alpha \mathrm{Gb}\sqrt{\rho }$$

where M, α, G, and b are the Taylor factor, a constant, the shear modulus and the Burgers vector, respectively. The corresponding values, M = 2.75, α = 0.166, G = 78 GPa, and b = 2.48 Å, were taken from Ref. [35]. As the heating rate increased from 0.3 °C/s to 200 °C/s, dislocation strengthening increased by about 40 MPa. Overall, fine-grain strengthening and dislocation strengthening can increase the hardness by approximately 72 HV. The calculated increase in hardness is higher than the actual measured increase in hardness. This is due to the decrease in solid solution strengthening and the increase in austenite volume fraction, resulting in a decrease in hardness. As shown in Figure 13, the hardness of GP is higher than that of quenched martensite at high heating rates. This is because the thin film RA and lath martensite in GP are finer, while the block RA in quenched martensite is larger. By observing Figure 11, it can be determined that the finer grains and higher dislocation density in GP are still the main reasons for its higher hardness. However, the detailed values and reasons for the local hardness increment are still a topic that needs to be discussed in the future. In summary, the higher hardness and finer RA film of this material exhibit better comprehensive mechanical properties, which makes it a promising raw material for wear-resistant components and automotive industry products.

### 4.3. Dynamic Analysis of Phase Transition during the Rapid Heating Stage

**Figure 15:**
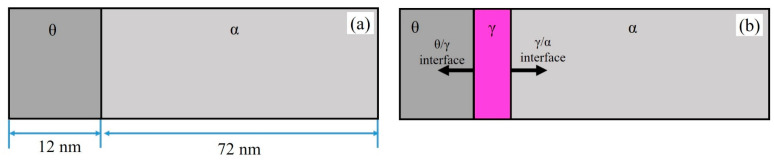
Hypothetical lamellar pearlite model: initial pearlite state (**a**), and austenite growth in pearlite (**b**).

To explain the reason for the formation of GP, the process of heating at 10 °C/s was selected for simulation. The growth process of austenite during heating and insulation stages was simulated by Thermo-calc software (2023a) DICTRA module, using the TCFE10 thermodynamic database and the MOBFE5 mobility database. For the work of the simulation, it was assumed that the shape of the pearlite was completely layered. A linear system, maintaining a cementite (θ)-to-ferrite (α) ratio of 1:6, was employed using a one-dimensional plane model. The use of one-dimensional models simplifies the phase transition process, and currently the main focus of inquiry is studying the dynamic reasons for the formation of heterostructures at high heating rates. Therefore, the use of the one-dimensional model of DICTRA to study the austenitization process of pearlite is an effective method and widely used [36,37]. The initial half-widths of cementite and ferrite were approximately 12 nm and 72 nm, respectively (Figure 15a), as observed by TEM. The simulation started at an initial temperature of 23 °C. The initial chemical composition attributed to cementite and ferrite was established based on insights derived from TEM-EDS analysis. In Figure 15a, the span of 12 nm marks the cementite domain (from 0 to 12 nm on the X-axis), while the residual expanse of 72 nm (from 12 to 84 nm on the X-axis) corresponds to the ferrite segment. As shown in Figure 15b, it is assumed that austenite nucleates and grows at the θ/α phase interface [10,38].

**Figure 16:**
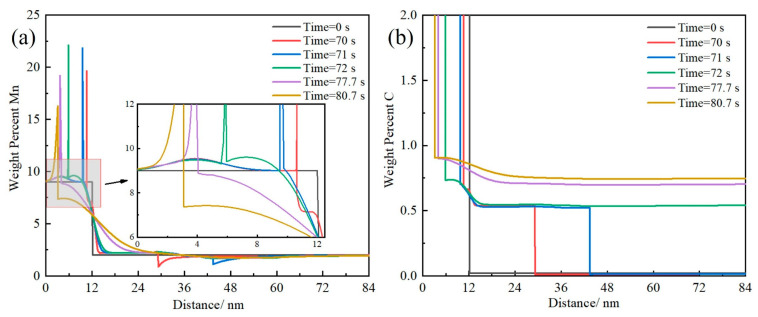
Evolution of Mn (**a**) and C (**b**) profiles during continuous heating at 10 °C/s and holding for 3 s. Solid lines of different colors represent different stages of heating and insulation.

Figure 16 shows the evolution of the C and Mn profiles during heating at 10 °C/s, with solid lines of different colors corresponding to each stage of the heating process. At 70–71 s (723–743 °C), θ/γ sharp peaks appear at the interface, and Mn grows very slowly towards the cementite. However, Mn in the ferrite diffuses into the newly formed austenite. This indicates that θ/γ interface is controlled by Mn diffusion (PLE mode), while γ/α interface migration is controlled by C diffusion (NPLE mode). At 71–77.5 s (743–800 °C), the θ/γ interface migration rate significantly increases, indicating that the phase transformation is controlled by C diffusion (NPLE mode). During the insulation stage (77.7–80.7 s), θ/γ interface movement rate decreases, and the area that has already been austenitized begins to undergo homogenization of Mn and C. The above results indicate that when heated at 10 °C/s, the phase transformation controlled by C diffusion is faster, while the diffusion of Mn is slower, resulting in a kinetic mismatch between rapid phase transformation and the slow diffusion of Mn. It is predicted that the appearance of an incomplete redistribution of Mn and a complete redistribution of C at the interface indicates the chemical boundary of Mn generation in the austenite. This chemical boundary will lead to the appearance of GP (Figure 7b).

When the heating rate is 0.3 °C/s, there is no heterogeneous distribution of Mn (Figure 10c). This is because the phase transformation is sequentially controlled by PLE, NPLE, and PLE at low heating rates, in which the PLE mode allows Mn to diffuse for a sufficiently long distance [39]. When the heterogeneity of Mn disappears, the initial morphology of pearlite is also difficult to preserve (Figure 7a). When the heating rate exceeded 10 °C/s, a heterogeneous distribution of Mn was obtained (Figure 10g,i). This is because the phase transformation under high heating rates is controlled by NPLE, and the interface movement rate is fast controlled by C, making it difficult for Mn to diffuse over a long distance [39]. Therefore, the higher Mn concentration in the cementite is inherited by austenite, which allows the Mn-riched film RA to be retained at room temperature.

## 5. Conclusions

1. As the heating rate increases, the PAGs were refined from 8.36 μm to 3.28 μm. This is due to the elimination of microstructural inheritance by the pearlite pretreatment. More austenite cores are obtained, through which only a few grains tend to grow, as the austenitization time shortens before the PAGs flatten.
2. As the heating rate increases, the total RA content increases from 14.98% to 21.17%, which is contributed by film RA. Film RA originates from the Mn-riched area of GP formed at high heating rates (>10 °C/s). A higher heating rate makes the chemical boundaries of Mn more distinct, which promotes the production of GP. GP consists of alternating Mn-riched RA and Mn-poored lath martensite.
3. As the heating rate increases, the hardness gradually increases from 628.1 HV to 663.3 HV, due to grain refinement and increased dislocation density. The higher hardness and finer RA film of this material exhibit better comprehensive mechanical properties, which makes it a promising raw material for wear-resistant components and automotive industry products.

## Figures and Tables

**Table 1:** Chemical composition of the tested steel (mass fraction, %).

C	Si	Mn	P	S	Mo	Fe
0.4	0.21	4.98	0.0035	0.0068	0.20	Bal.

**Table 2:** Dislocation density of tested steels at different heating rates.

Heating Rate/°C/s	0.3	1	10	50	100	200
Dislocation density/×10^12^ cm^−2^	1.3614	1.2855	1.3736	1.4179	1.4611	1.4832

## Data Availability

The original contributions presented in the study are included in the article, further inquiries can be directed to the corresponding author.
